# Perspectives on rapid fentanyl test strips as a harm reduction practice among young adults who use drugs: a qualitative study

**DOI:** 10.1186/s12954-018-0276-0

**Published:** 2019-01-08

**Authors:** Jacqueline E. Goldman, Katherine M. Waye, Kobe A. Periera, Maxwell S. Krieger, Jesse L. Yedinak, Brandon D. L. Marshall

**Affiliations:** Department of Epidemiology, Brown University School of Public Health, 121 South Main Street, Box G-S-121-2, Providence, RI 02912 USA

**Keywords:** Overdose, Opioids, Fentanyl, Harm reduction, Rapid test, Qualitative

## Abstract

**Background:**

In 2016, drug overdose deaths exceeded 64,000 in the United States, driven by a sixfold increase in deaths attributable to illicitly manufactured fentanyl. Rapid fentanyl test strips (FTS), used to detect fentanyl in illicit drugs, may help inform people who use drugs about their risk of fentanyl exposure prior to consumption. This qualitative study assessed perceptions of FTS among young adults.

**Methods:**

From May to September 2017, we recruited a convenience sample of 93 young adults in Rhode Island (age 18–35 years) with self-reported drug use in the past 30 days to participate in a pilot study aimed at better understanding perspectives of using take-home FTS for personal use. Participants completed a baseline quantitative survey, then completed a training to learn how to use the FTS. Participants then received ten FTS for personal use and were asked to return 2–4 weeks later to complete a brief quantitative and structured qualitative interview. Interviews were transcribed, coded, and double coded in NVivo (Version 11).

**Results:**

Of the 81 (87%) participants who returned for follow-up, the majority (*n* = 62, 77%) used at least one FTS, and of those, a majority found them to be useful and straightforward to use. Positive FTS results led some participants to alter their drug use behaviors, including discarding their drug supply, using with someone else, and keeping naloxone nearby. Participants also reported giving FTS to friends who they felt were at high risk for fentanyl exposure.

**Conclusion:**

These findings provide important perspectives on the use of FTS among young adults who use drugs. Given the high level of acceptability and behavioral changes reported by study participants, FTS may be a useful harm reduction intervention to reduce fentanyl overdose risk among this population.

**Trial registration:**

The study protocol is registered with the US National Library of Medicine, Identifier NCT03373825, 12/24/2017, registered retrospectively. https://clinicaltrials.gov/ct2/show/NCT03373825?id=NCT03373825&rank=1

## Introduction

Opioid overdose is an ongoing public health crisis in the United States (US), exacerbated by fatal overdoses involving illicitly manufactured fentanyl (herein referred to as “fentanyl”), a powerful synthetic opioid contaminating the North American drug supply [1, 2]. As early as 2013, several states in the US, particularly those in the Northeast, began to report a rise in the total number of overdose deaths attributable to fentanyl and related analogs [3, 4]. By March 2015, due to the rapid increase in fentanyl-involved overdose fatalities, the US issued a nationwide alert to law enforcement identifying the dangers and increased prevalence of fentanyl in the illicit drugs supply [3–5]. Between 2015 and 2016, there was a 21% increase in the age-adjusted rate of overdose deaths in the US, driven by a rise in deaths involving synthetic opioids, primarily that of fentanyl [6, 7]. These overdose fatality trends highlight the urgent need to address fentanyl contamination in the US drug supply and its associated harms.

The rate of fentanyl-attributable fatal overdose has been particularly high among residents of New England, the northeastern region of the US [8, 9]. In 2016, fentanyl was found in 58% of all overdose deaths in Rhode Island, a state in New England with an overdose death rate about 1.5 times greater than the national average [10, 11]. Additionally, fatal opioid overdoses are affecting younger populations than previous years. In Rhode Island, young adults have the fastest growing rate of fatal overdoses; more than one in four fatal fentanyl-related overdoses in 2016 were among people between the ages of 18 and 29 years [12, 13].

When implemented effectively, harm reduction interventions can reduce the rate of fatal opioid overdoses among people who use drugs [14, 15]. For example, the distribution of naloxone (an opioid antagonist that reverses the effects of an opioid overdose and can be easily used by laypeople) through community- and pharmacy-based overdose education and naloxone distribution (OEND) programs has been shown to reduce opioid overdose mortality [16–18]. Communities with OEND programs have shown greater reductions in overdose mortality compared to those without such programs [16, 17]. Moreover, the distribution of naloxone to laypersons has been found to be cost-effective [16, 17, 19]. However, due to the increased and varying potency of fentanyl and related analogs, naloxone doses previously adequate to reverse an opioid overdose may not be consistently sufficient [20]. In addition, consumption of fentanyl causes more profound respiratory depression than other opioids and produces clinically distinct symptoms (e.g., bradycardia, chest wall rigidity) that precipitate rapid onset of overdose death, narrowing the window of opportunity to administer naloxone [21, 22]. Due to the drug’s potency, only a miniscule amount—the equivalent of several grains of salt—can cause an overdose death [23] and can be nearly impossible to identify in illicit drugs with the naked eye.

Rapid fentanyl test strips (FTS) represent an emerging harm reduction intervention that may help to prevent unintentional fentanyl exposure and accidental opioid overdose. These tests have the ability to detect the presence of fentanyl and some analogs in urine or in drug samples dissolved in water that are believed to be contaminated [24, 25]. Drug checking has become a staple harm reduction intervention in parts of Europe and Canada, through the establishment of drug testing programs and supervised injection facilities (SIFs) [24, 26–28]. People who use drugs (PWUD) living in parts of Europe and Canada can bring their drugs to harm reduction organizations or SIFs to have them tested for adulteration; however, PWUD in the US do not have access to the same types of programs because of legal barriers to implementing SIFs at the federal level [29–31]. In studies of fentanyl testing done outside of the US, persons who use FTS and who receive a positive test result may be more likely to partake in overdose prevention strategies than a person who is not aware that their drug is contaminated with fentanyl [32, 33]. Therefore, the use of FTS may be an important harm reduction practice to inform and increase engagement in overdose prevention behaviors, employed before an overdose occurs.

In the US, where SIFs have not been legalized, FTS offer PWUD the option to test their own drugs in a private setting. Because evidence for distributing FTS for home use by PWUD is nascent, there are many uncertainties regarding the efficacy and the safety of FTS self-testing as a means of overdose prevention [34]. Preliminary research has found mixed results regarding the efficacy and acceptability of fentanyl self-testing as harm reduction strategy. A study performed in North Carolina among people who injected drugs found FTS were widely used among the sample and that a positive FTS resulted in changes in drug use behavior [35]. Other studies conducted in Canada have found that people who were using supervised injection facilities did not use FTS frequently because of commonly held perceptions that most drugs were fentanyl-contaminated, and therefore, participants did not need to confirm its presence [34, 36]. This conflicting evidence points to the fact that further research is needed to understand the ways in which FTS are viewed and used by PWUD. The need for additional research is particularly urgent as FTS are being distributed by multiple US harm reduction organizations and state departments of health for at-home use [37–42]. In Rhode Island, organizations have recently started distributing FTS as part of local overdose awareness events [43].

Despite growing interest in fentanyl drug checking technology, perceptions of and attitudes towards FTS as a tool to reduce overdose risk among young PWUD have not been investigated in the US. Therefore, the aims of this study were to (1) understand perspectives on take-home FTS among young adults (age 18–35) who use drugs in Rhode Island and (2) determine whether positive results from the FTS led to behavioral changes in the way that people use drugs. Understanding the perceptions of PWUD regarding FTS can formatively assist in the development and implementation of rapid fentanyl testing programs in the US and in other settings experiencing a high burden of fentanyl-involved overdose.

## Methods

### Participant recruitment

From May to September 2017, young PWUD were invited to participate in a pilot study designed to understand perceptions of take-home FTS among young adults who use drugs. For this study, eligibility criteria were informed by a recent investigation of fentanyl-involved overdose deaths in Rhode Island [13]. Specific eligibility criteria included (1) being 18–35 years of age; (2) currently living in Rhode Island; (3) being able to speak English; (4) reporting the use of heroin, cocaine, purchasing prescription pills on the street, or injecting any drug in the last 30 days prior to study enrollment report; and (5) being able to provide informed consent. Many of the participants were recruited from online classifieds (i.e., Craigslist) which discussed the eligibility criteria for our study, and by word of mouth from peers who participated in the study. Additionally, fliers were placed in public areas where PWUD are known to congregate, such as bus stations, transit centers, and local universities. This study was approved by the Brown University Institutional Review Board (#1612001662). The study’s protocol is also documented on ClinicalTrials.gov (Identifier NCT03373825).

### Intervention protocol

**Fig. 1:**
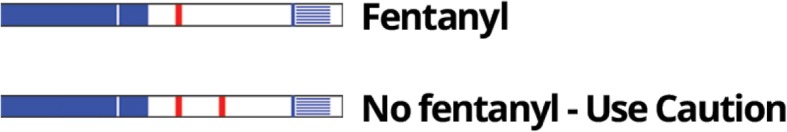
Fentanyl rapid test strip results label. This is the key that was placed on each of the FTS packages to assist with interpretation of the test results. This key shows that a strip with one red line indicates a positive test and a strip with two red lines indicates that the test is negative, but that the participant should still use caution

At the initial baseline visit, all consenting participants completed an hour-long survey, performed by trained research assistants, which collected information on participant demographics (such as race, gender, age, educational attainment, housing and employment status), drug use patterns, and overdose history. The protocol for the initial baseline visit has been described in detail elsewhere [44]. Once the survey was completed, participants were shown two brief instructional videos demonstrating how to use and interpret BTNX Inc. Rapid Response™ Fentanyl Test Strips [45]. These FTS are disposable, single-use immunoassay tests with a detection level of 20 mg/ml; the tests provide a binary result of either positive or negative for the presence of fentanyl. The tests have high sensitivity and specificity for detecting fentanyl and some analogs [46]. Further, all participants were given a handout that was written in plain language and discussed how to test urine or powdered drugs and pills with the FTS. Additionally, we placed a key on each of the FTS packages to assist with interpretation of the test results (Fig. 1. Fentanyl rapid test strip results label).

From May 2017 to mid-July 2017, the first 40 participants were instructed to use the FTS to test their urine (after drug use). We instructed participants in this first group to use the FTS in their urine in concordance with how the product is intended to be used [25, 45]. During the recruitment period, a separate study found that the FTS retained high specificity and sensitivity when used to test drug residue from bags, spoons, or pills crushed in water [46]; therefore, the study protocol was amended to instruct the second group of participants to test a drug sample or drug residue dissolved in water (prior to consumption) [46]. Participants recruited between mid-July 2017 and September 2017 were thus trained to test a drug sample or their drug residue (before consumption). In both groups, participants were instructed that a negative FTS did not necessarily indicate an absence of fentanyl contaminants or zero overdose risk and were instructed to always use their drugs with caution. Participants were provided with overdose education and naloxone following the FTS training. This education also included training on how to use drugs more safely (such as using with someone else, having naloxone, and using a smaller initial dose). Once the training was complete, participants were subsequently asked if they felt comfortable using and interpreting FTS independently or had any concerns regarding the previously described training materials and were then given ten BTNX Rapid Response™ Fentanyl Testing Strips for personal use.

### Data collection

At each participant’s follow-up visit, occurring approximately 2 to 4 weeks after baseline enrollment, consenting participants completed a brief, researcher-administered quantitative survey, as well as a structured qualitative interview designed to capture attitudes regarding FTS use. Interviews were conducted by trained research assistants and were recorded for subsequent transcription. Research assistants had a guide of 12 open-ended questions that explored participants’ test use and experience. The questions explored FTS use and general opinions of the tests by asking, “Did you use any of the tests?” and “How was that experience for you?”. Additionally, questions, such as “What was not useful about the fentanyl tests?”, “What got in the way of using the tests?”, and “Is there anything that would make it easier to use the test?”, brought forth discussions about barriers to FTS use. The prompt, “Based on the test strip results, did you do anything differently when it came to how you used your drugs?”, elicited discussions regarding behavior change as a result of FTS use. Qualitative interviews lasted between 10 and 20 min. Participants were paid $25 after their initial visit and $50 after completing the follow-up visit.

### Data analysis

Interviews were transcribed by four members of the research team. The data analysis was performed by using a thematic analytic approach, an iterative process of repeatedly analyzing data in order to recognize categories that subsequently become the themes of a research study [47]. For this study, a deductive approach was utilized in order to develop an initial coding scheme, which was constructed from the interview template [48]. A code book was then developed and refined as new ideas and themes were discovered in the transcripts. Subsequently, an inductive approach was utilized, which brought forth new concepts and themes from the reading of the interview transcript [49]. A final coding template was created and agreed upon. Six interviews were cross-coded by two research team members to ensure a concordance with a kappa of at least 80%. Cross-coding occurred to prevent bias and to ensure uniform use of the coding template by the two coders. Descriptive statistics of the population were drawn from responses to the baseline researcher-administered survey. NVivo (version 11) was utilized for qualitative data retrieval and management.

## Results

Of 93 recruited participants, 81 (87%) returned for follow-up and were included in this analysis. A summary of the participant characteristics is shown in Table 1. The mean age was 26 (SD = 4.7), 45 (55.5%) were male, and the majority (*n* = 45, 55%) identified as white. Over half of the participants (*n* = 55, 68%) expressed concern about their drugs being contaminated with fentanyl at baseline.

**Table 1:** Study population characteristics (*n* = 81)

Characteristic	*N* (%)
Age	
Mean (SD)	26. 5 (4.7)
Sex	
Male	45 (55.5)
Female	33 (40.7)
Non-binary	3 (3.7)
Race	
White	45 (55.5)
Black	12 (14.8)
Other/mixed race	24 (29.6)
Ethnicity	
Non-Hispanic	62 (76.5)
Hispanic	19 (23.5)
Educational attainment	
Less than 12th grade	8 (9.9)
High school diploma or higher	73 (90.1)
History of homelessness	
Yes	48 (59.3)
No	33 (40.7)
Drug used in the 30 days preceding study^a^	
Prescription pills purchased on the street	50 (61.7)
Heroin	34 (42.0)
Cocaine	60 (74.1)
Injection drug use (any drug)	35 (43.2)
Ever experienced an overdose	
Yes	52 (64.2)
No	29 (35.8)
Ever experienced an overdose from a drug suspected to contain fentanyl	
Yes	15 (18.5)
No	66 (81.5)

^a^These categories are not mutually exclusive

Of those who used at least one FTS, 37% reported regular heroin use, 24% reported regular cocaine use, 13% reported regular non-medical prescription pills use, 47% reported lifetime injection drug use, and 50% received at least one positive FTS result (Table 2). A greater percentage of those who were in the urine testing group reported cocaine use compared to those in the residue testing group (49% vs. 24%); otherwise, few baseline characteristics differed between these two groups. Additionally, about half of the participants from each group who received a positive test result reported altering the way they use drugs. Compared to the residue testing group, a greater proportion of those in the urine testing group reported changing their behavior following a positive FTS (62% vs. 38%); however, there were no significant differences in the specific behaviors that people reported changing between groups. A further quantitative analysis measuring the use of FTS among this sample has been detailed elsewhere [50].

**Table 2:** Fentanyl rapid test strip use among young adults who use drugs in Rhode Island (*n* = 81)

Characteristic	*N* (%)
Participants who used at least one test strip	62 (76.5)
Of the participants who used at least one FTS (*n* = 62), participants reported^a^:	
Regular heroin use	23 (37.1)
Regular cocaine use	24 (38.7)
Non-medical prescription pill use	13 (21.0)
Lifetime injection drug use	29 (46.7)
Of the participants who used at least one FTS (*n* = 62), the number (proportion) who received at least one positive FTS	31 (50.0)
Of the who received at least one positive FTS (*n* = 31), participants reported altering the way they used drugs^a^:	
Used less	14 (45.2)
Used with someone else around	12 (38.7)
Went slower	13 (41.9)
Did a tester	11 (35.5)
Threw them out	3 (9.7)
Sold them	3 (9.7)
Gave them away	2 (6.5)

^a^These categories are not mutually exclusive

Overall, the majority of participants who used FTS expressed positive opinions regarding the utility and simplicity of the tests. Five key themes emerge from the interviews: (1) FTS were a tool to confirm suspicions of fentanyl adulteration, (2) differences in ease of FTS testing depended on testing method, (3) participants re-distributed tests to people with high perceived overdose risk, (4) participants preferred testing their drugs in private, and (5) presence of fentanyl led to self-reported behavior change.

### FTS were a tool to confirm suspicions of fentanyl adulteration

In general, participants expressed positive opinions regarding FTS, stating that they were easy to use and that they provided valuable information regarding the presence or absence of fentanyl in a drug sample. Respondents stated that the FTS were useful especially when a drug supply source was not trusted by the participants.

Everything was useful. Those tests opened my eyes, and it has saved my life, and I can gladly say I haven’t taken any more because I was going to take two bags. If I had took those two bags, I think I wasn’t even going to be here right now (Respondent 39, male of non-disclosed race, age 28, residue testing group).

One respondent proceeded to not use his drugs because of fear of overdose. Like other respondents, knowledge of fentanyl adulteration led to fentanyl avoidance.

But it’s (fentanyl) going to show up in the test, so it is kind of worth it. That’s what I’m saying is, you could save your life by using this. Or you could not use it and do what you’re going to do and be dead...I thought it came out positive, so I got rid of the fentanyl (Respondent 17, white male, age 20, urine testing group).

Another respondent provides an example of how he used FTS to avoid consuming of fentanyl adulterated drugs.

I contacted a local dealer of mine that I had gone to in the past, that I had mentioned before, I was like questionable of his product, so I told him that I had these strips and that I was going to test, to test his stuff for fentanyl to see if it was good or not and showed him the positive result… with those tests, I was able to do that with a couple other dealers between now and then to root out my chances of getting a tainted product (Respondent 54, White male, age 23, residue testing group ).

This participant, along with others, discussed how some dealers were unaware or non-forthcoming regarding fentanyl adulteration. The participant also suggested that he sought out dealers whose drugs were not contaminated with fentanyl.

### Differences in ease of FTS testing depended on testing method

When prompted about barriers to using FTS, participants from both testing groups expressed that using the FTS were “straightforward” and “easy to do.” A majority of participants expressed positive opinions of the accessibility of FTS use.

They’re very useful, very straightforward, very simple. I think it’s a great tool, I think they should be given out on a street corner (Respondent 11, white male, age 29, urine testing group).

I mean the packaging was easy, I mean it’s pretty discreet…they weren’t inconvenient, they weren’t hard to use, they weren’t, you know, embarrassing to be seen with or anything (Respondent 36, white female, age 34, residue testing group).

Another participant reported FTS to be easy to use and discussed showing others how to use FTS.

It was easy, you know, very easy to do in front of them. Very easy to explain. When I say in front of them, I mean the dealers or whoever I was sourcing the product from... It was easy to do it in front of them, show them how it works, show them that it’s effective even with just the residue in the baggie (Respondent 54, white male, age 23, residue testing group).

Participants in the urine group expressed wanting to be able to test their drugs ahead of time in order to prevent an overdose.

If there was a like test strip you know that you could mix a bit of your heroin, or what you think is heroin in water and dip the strip in it, and something like that, you know, like to be able to test it before you use it. Because, you know, afterwards it could be too late, you know (Respondent 13, male of non-disclosed race, age 22).

In addition to wanting to use FTS before drug use, several participants alluded to the fact that using a urine-based test was not convenient.

I didn’t have to pee all the time so like, sometimes when I wanted to take it I would just have to drink a bunch of water but, it worked out (Respondent 27, female of an unspecified race, age 25, urine testing group).

### Participants distributed tests to people with high perceived overdose risk

Some participants from both groups described engaging in “secondary distribution” of FTS, that is, the distribution of FTS to people in the participant’s networks, such as friends, family members, and casual acquaintances who they perceived as having a higher risk for using a drug contaminated with fentanyl:

I gave them to close friends of mine who I suspect still use and casual friends of mine...and you know I just wanted them to know, ‘hey listen, you can test if it’s fentanyl with this, I don’t know if it’s a good thing or a bad thing, but you can test’ (Respondent 10, black male, age 30, urine testing group).

Well one of my one of my aunts has, I don’t see her very much, but I know that she’s had like a history with addiction, so I figured I give her one and just let her know about what it does pretty much (Respondent 59, white female, age 22, residue testing group).

Another participant described handing out 5–6 of her test strips to people she knew from the methadone clinic, who had previously mentioned wanting to know if fentanyl was in their drug supply.

They were not just close friends, but a couple of people I met at the clinic and stuff, and they actually were people who really want to know if the fentanyl is actually in the drugs they are using, and they actually use a little bit more than I do. On a daily basis they still use, so I felt like it was very important to have (Respondent 55, white female, age 28, residue testing group).

### Participants preferred testing their drugs in private

When participants were asked how it felt to test their drugs at home, followed by questions that assessed their willingness to go to a local health organization to get their drugs tested, a majority of participants expressed that they preferred to use FTS at home. Participants reported two primary reasons for wanting to test at home. Some reported they would rather use drug tests at home in order to avoid feeling judged by others.

I think that people like to test their own stuff in private. I have friends that would get the drug test kits from the stores and just test in private because like a lot of stigma attached to things. So when you go to a place where you have to do drug testing even the people there can kinda look at you kinda weird um no matter what their reason is (Respondent 2, multiracial female, age 29, urine testing group).

In addition to reports of feeling judged or stigmatized, other participants suggested fear of legal ramifications or other risks if they were to test their drugs someplace other than their home.

I would rather be at home. I wouldn’t want to be taking my drugs into somewhere, and it’s just like, a lot of people would feel that way too, because like I know damn well that I’m nothing. I’m just a statistic. But a lot of people think that they would go to jail I’m sure if they went to a place and they said, hey test my drugs (Respondent 11, white male, age 29, urine testing group).

Just because it’s more private, it’s in my house, I wouldn’t have to risk getting caught by the police bringing it somewhere. I would just, I don’t know, I would never bring it somewhere to get it tested honestly, never (Respondent 81, white female, age 25, residue testing group)

I feel like going to a local health organization it would just be, I hate to say this, an opportunity for the cops to try to stop you as you’re walking in to have it tested. Definitely at home, I would definitely do it at home where it’s more secure and I don’t have to worry about anything else (Respondent 37, white female, age 28, residue testing group).

### Presence of fentanyl led to behavior change

During the qualitative interview, participants described behaviors such as using a “tester” (i.e., an initial small dose of the drug to determine potency), having naloxone nearby, using a drug with other people around, and disposing of the drug, particularly as a result of receiving a positive test. Here, participants describe how they would use an initial smaller dose of the drug they tested, a “test shot,” in reaction to a positive FTS result. Though this participant was not using drugs during the study period, she advised a friend who was using drugs to go slower because of the positive FTS.

A friend of mine was shooting up and before they did that I said let me test it, so I grabbed the cap after they used it and I tested it, and it was positive for fentanyl. And they, asked me what exactly fentanyl does and I said it’s way stronger than your heroin and it has the potential to kill you with a drop. And they were like “what am I supposed to do” I said, honestly, you shouldn’t take that, but I know you’re going to, so take it in portions...instead of putting the whole .4 to the face, they would do .1 at a time, and, you know, with a little bit of time in between, in between, each one (Respondent 68, multiracial male, age 21, residue testing group).

I always did them [FTS] with someone else. It was me and two of my friends a few times. And the stuff they were getting, they kept assuming it had fentanyl in it, but they weren’t 100% sure. Usually when heroin comes out white or clear it’s fentanyl. So, when we tested it and it was positive for it, it made them, you know, they did a tester shot before they did the rest and that was smart on their end because it was really strong, and they would have worn out otherwise (Respondent 61, white female, age 35, residue testing group).

Another participant described how a positive FTS result contributed to a change in her “mindset” about her drug use and keeping naloxone nearby.

I had Narcan and I had it in the room, like I literally had it on the couch next to me, cause it, it honestly, just like thinking fentanyl in it was one thing, but like knowing it was in it kind of like changed my, like my mindset going into it (Respondent 59, white female, age 22, residue testing group).

Similar to other participants, another respondent described her response to a positive FTS result. She describes her conversation with her cousin, a dealer, about disposing of a significant amount of fentanyl-contaminated heroin.

I used one for myself, and then, the other nine I gave to my cousin who is a drug dealer, um, and I told him to give me nine samples of his dope, and I followed the video that I watched … And out of the nine that came back, seven were positive for fentanyl. So, I told him, you either have seven murder charges on your record, and you have the rest of your life in prison, you don’t get to see your kids get married, graduate. Kinda give him a little guilt trip on it, but I convinced him to flush it. He had almost $2000 worth of fentanyl laced heroin and he got rid of it (Respondent 68, biracial female, age 26, residue testing group).

Finally, participants who used the FTS after drug use described various overdose prevention strategies for the *next time* they used drugs following a positive FTS, including warning others about fentanyl contamination following a positive FTS.

But being able to relay, relay that information to people, that it actually would impact in ways that hopefully would change some of their actions and how they conducted, either, using or, giving it to other people (Respondent 23, biracial male, age 25, urine testing group).

I would say we were definitely a lot more cautious about what we were doing, like definitely a lot more ready for something to, you know, go wrong...I definitely, like, would pace myself a lot slower with the drugs. And you know, it was like I said, it’s kind of sad to say but we were almost expecting an overdose or such. And so, if that did happen, you know like at least somebody could be like, oh and jump on it and act fast (Respondent 22, white male, age 22 , urine testing group).

## Discussion

The results of this study demonstrate that many young PWUD at risk of a fentanyl overdose perceive FTS as a feasible and acceptable harm reduction tool. In this study, we assessed two different applications of FTS (urine testing and residue testing) and found that residue testing is more convenient and allowed for participants to know about fentanyl adulteration before drugs are consumed. The majority of participants suggested that FTS were straightforward to use and did not cite significant barriers to use. After receiving a positive test result, many participants described precautions that they believed would prevent an overdose, such as using a drug with others around, keeping naloxone nearby, or using a tester. These findings suggest FTS may represent an important harm reduction intervention for opioid overdose prevention among young adult PWUD.

While there are other methods confirming the presence of fentanyl in one’s drug supply, such as the use of Raman spectroscopy and FTIR spectroscopy, prior research has found that the BTNX Inc. Rapid Response™ Fentanyl Testing Strips have higher specificity and sensitivity and are significantly less expensive than other methods [46]. Nonetheless, testing illicit drugs, through either FTS or chemical analyses, has proven to be an effective approach in identifying adulterants that pose added overdose risk to PWUD, as indicated by ongoing drug checking efforts in Canada and in European countries [24, 27].

In places where drug testing is legal, such as Canada and Europe, people who want to have their drugs tested most often need to bring their substances to specific locations. For instance, SIFs in Canada distribute FTS to their clients; however, clients must test their drugs in that same setting [51–53]. In Europe, drug testing largely takes place at music venues and mobile test sites with trained professionals [27, 54]. In contrast, syringe service programs (SSPs) in the US are just beginning to disseminate FTS to their clients for at-home use outside of a clinical or supervised context [40, 41]. State governments and departments of health, such as those in California, Vermont, and Maine, are also providing funding for the purchase and dispersal of take-home FTS through existing SSPs [37–39, 42]. However, there are debates as to whether there is sufficient evidence to support the effectiveness of home testing programs [32]. Given that FTS have the potential to return false negative results, PWUD using FTS may proceed to use their drugs without added precautions to prevent fentanyl overdose risk even if their drugs are contaminated with fentanyl. Outside of monitored environments such as at SIF, a false negative test in a private setting could lead to a higher risk of overdose [34]. To mitigate this concern, all participants were instructed during the FTS training that false negatives are possible and that a negative result does not necessarily mean an absence of overdose risk. Private use of FTS (i.e., outside of monitored environments) may represent a novel harm reduction strategy to reduce the risk of fentanyl-related overdose, even though concerns persist regarding the risk for unintentional overdose resulting from a false negative [34].

Harm reduction technologies used in private settings have appeal to PWUD in the US who fear the legal ramifications and stigma associated with use of harm reduction services, such as SSPs [55, 56]. In agreement with US studies that have found that harm reduction service can be uncomfortable and unapproachable for some PWUD, particularly for young adults, our participants recounted their reluctance to engage in FTS use at professional agencies or harm reduction organizations [57–59]. Our study suggests that young PWUD are comfortable using FTS on their own and would prefer to use them either in their own home or in another private setting due to concerns for privacy and fear of arrest and facing stigma from the public, found to be a significant barrier to harm reduction uptake in earlier studies [58]. Furthermore, secondary distribution of FTS, mirroring documented occurrences of secondary distribution of sterile syringes, has the potential to benefit PWUD who are either uncomfortable accessing or are not closely engaged to healthcare services [60, 61]. Collectively, these findings indicate that interventions which permit drug checking in private environments may potentially increase accessibility and acceptability of drug testing among young PWUD. Additionally, our participants may have used take-home FTS as this intervention allows for self-efficacy and peer-to-peer interaction, which have been found to lead to successful implementation harm reduction programs in previous research [57].

Upon receiving a positive FTS result, many participants were motivated to engage in various harm reduction techniques, including using a smaller dose, having naloxone nearby, using the drug with someone else around, or disposing of their drugs entirely. Consistent with another study of FTS conducted in the US [35], participants stated they employed these precautions because they were made aware of fentanyl contamination. Prior studies of self-testing technologies suggest similar results—that is, rapid self-testing may contribute to an increase in harm reduction behaviors. Multiple studies on rapid self-testing for HIV, a technology that was legalized for at-home use in the US in 2012, have reported noticeable increases in both perceptions of risk and target risk reduction behaviors [62–65]. Additionally, studies have shown HIV self-testing is a successful intervention for increasing routine HIV testing among hard to reach and hard to engage populations, such as young adults engaging in high-risk behaviors [66, 67]. Such findings offer promise for rapid testing technology as a key component of harm reduction interventions for fentanyl overdose. Furthermore, in Canadian studies of FTS, and in initial studies of FTS in New York City, participants who received a positive FTS result changed their behavior in similar ways to the current study; they slowed down their use, used a smaller dose, or disposed of the drug that was found to contain fentanyl [25, 26, 42]. Given these results, FTS should be explored as an additional means of preventing opioid overdose used in tandem with other harm reduction measures, such as naloxone distribution and overdose education. In contrast, it has been hypothesized that in areas where fentanyl contamination is pervasive, PWUD who have taken drugs that contain fentanyl and have not experienced an overdose may become complacent in their use of overdose prevention strategies [34]. This could prove to be true in Rhode Island where participants noted that fentanyl contamination is likely. Ultimately, future research is needed to evaluate FTS interventions to understand how FTS may contribute to behavior change among young adults.

This study had a number of limitations. First, as the average follow-up time frame was less than a month, some participants did not have an opportunity to try the FTS, given that some reported a lack of opportunity to either buy or use the drugs in the study’s timeframe. Many of the participants who had not used FTS during the study had expressed that given a longer time frame for use, they would have tried the FTS. Future research regarding fentanyl FTS should include a longer period between the provision of the FTS and follow-up. Second, though interviews varied in length, they generally did not last more than 15 min. Beyond this pilot project, future studies could conduct longer interviews with participants, which may allow for a more nuanced understanding of the influence and effect of FTS utilization on behavior change among young PWUD. Third, discussions of how drug use changed following a positive FTS result could be affected by social desirability bias. Additionally, selection bias may have occurred due to healthy screenee bias [68], in which PWUD who want to avoid fentanyl may be more likely to enroll in a study of FTS. Nonetheless, our results suggest that participants altered their drug behavior as a result of having a definitive knowledge that their drug contained fentanyl. Fourth, while we ascertained that many of the participants had a history of homelessness, we did not ask if participants faced challenges of using FTS due to current housing instability or homelessness. Therefore, we cannot make claims about FTS usability among those currently experiencing homelessness. Finally, this study took place in Rhode Island, a state with a high burden of fentanyl-related overdoses and fentanyl contamination. As such, results may not be generalizable to other settings, particularly those in which the presence of fentanyl contamination in illicit drugs is less common.

## Conclusion

In sum, FTS may prove to be an important harm reduction tool, particularly in the context of the US opioid overdose crisis, as fentanyl and fentanyl analogs are increasingly responsible for the rising rate of overdose fatalities in the US [6]. This study is among the first to explore the perceptions and use of FTS among young adults who use drugs. Participants expressed that FTS were an efficient and straightforward tool, and many reported distributing them to other persons in their social networks. Participants also discussed the value of FTS as a harm reduction tool for identifying fentanyl contamination and informing overdose prevention behaviors. Take home rapid tests that have the ability to detect the presence of fentanyl and other analogs may offer PWUD additional options for overdose prevention in the face of increasing contamination in the US drug supply.

## Abbreviations

FTS: Rapid fentanyl test strips
HIV: Human immunodeficiency virus
OEND: Overdose education and naloxone distribution
PWUD: People who use drugs
SIF: Supervised injection facility
SSP: Syringe service program
US: United States
